# Association between disseminated cancer and postoperative 30-day mortality in adult patients with brain tumor craniotomy

**DOI:** 10.3389/fonc.2025.1555850

**Published:** 2025-12-19

**Authors:** Yufei Liu, Ke Cao, Rui He, Wenjian Zheng, Zongyang Li, Xuanchen Li, Mengqi Wang, Haofei Hu, Guodong Huang

**Affiliations:** 1 Department of Neurosurgery, Shenzhen Key Laboratory of Neurosurgery, the First Affiliated Hospital of Shenzhen University, Shenzhen Second People’s Hospital, Shenzhen, Guangdong, China; 2 Brain Injury Centre, Department of Neurosurgery, Renji Hospital, Shanghai Jiao Tong University School of Medicine, Shanghai, China; 3 Nephrological Department, Shenzhen Second People’s Hospital, The First Affiliated Hospital of Shenzhen University, Shenzhen, Guangdong, China

**Keywords:** disseminated cancer, brain tumor, craniotomy, propensity score matching, mortality

## Abstract

**Background:**

Quantitative evidence on the association between disseminated cancer (DC) and 30-day postoperative mortality after tumor resection craniotomy in adults is limited. This study evaluates the association between them.

**Materials and methods:**

This retrospective analysis utilized propensity score matching (PSM) on cases extracted from the American College of Surgeons National Surgical Quality Improvement Program database (2012-2015). The study examined DC as the independent variable and 30-day postoperative mortality as the dependent outcome. A logistic regression analysis was conducted on the PSM data that were 1:1 matched. The DC-mortality association was assessed using robust statistical estimation methods.

**Results:**

The study cohort comprised 18,642 eligible patients (52.6% male, 47.4% female), including 4,022 (21.57%) with DC. The mortality rate was significantly higher in DC patients (4.97%) compared to the overall cohort undergoing tumor-related craniotomy (2.46%). Multivariate analysis and propensity score-adjusted analysis demonstrated that, compared with non-DC, the postoperative 30-day mortality of patients with DC undergoing craniotomy for brain tumors significantly increased, with associated odds ratios of 1.72 to 2.06.

**Conclusion:**

Given the relatively high risk of mortality within 30 days after craniotomy in patients with DC, both preoperative surgical decision-making and postoperative management strategies should be appropriately modified to reduce mortality.

## Background

Craniotomy is the cornerstone of most brain tumor treatments around the world. It is unfortunate that craniotomies for brain tumors displays significant complications and mortality (1, 2). Postoperative 30-day mortality, also known as 30-day postoperative mortality, is widely used to assess the prognosis of patients undergoing surgery (3, 4), including brain tumor surgery (5, 6). Patients with disseminated cancer at higher risk for postoperative mortality see improved prognosis with changing clinical management (7). Previous studies have been conducted on the relationship between brain DCs and surgical outcomes. Surgical resection is generally beneficial in selected patients with brain metastases (8, 9), even locally recurrent brain metastasis (10). Surgery is not only the means of obtaining a histopathological diagnosis, but also the effective treatment for rapid relief of complications or life-threatening neurological symptoms associated with significant mass effect, hydrocephalus, and peritumoral edema (11, 12). Local aggressive surgical resection is reasonable in patients with limited intracranial disease, controlled primary disease, and high performance status (13). Surgical intervention can prolong the survival of liver cancer (14), lung cancer (15) and gastrointestinal cancer (16) patients with brain metastases. However, Surgical intervention among patients with disseminated malignancy carries substantial postoperative complications, morbidity or mortality (17–19). Notably, a multidisciplinary approach is strongly recommended to personalize the treatment (includes surgery, stereotactic radiosurgery, immunotherapies or targeted therapies) of each patient with brain metastases (20). Our previous study identified DC as one of the six predictors of a prognostic model that predicts postoperative 30-day mortality for brain tumors (21). Hence, clarifying the clear relationship between brain metastases and postoperative 30-day mortality after craniotomy could provide preoperative guidance and optimize management strategies.

Previous studies have mainly applied traditional regression models based on analytical adjustments to control for confounders, which could hinder the investigation of the link between DC and prognosis (14, 22–24). However, propensity score matching (PSM) is an increasingly popular method to adjust for confounding in observational studies (25–27), including brain tumor research (28–31). Doctors and patients with DC, as well as their families, often struggle to make a choice between surgical treatment and conservative therapy. Hence, To investigate the DC-postoperative 30-day mortality association, we performed a propensity score-matched retrospective analysis using data from adult patients undergoing craniotomy for tumor resection.

## Participants and methods

### Data source

American College of Surgeons National Surgical Quality Improvement Program (ACS-NSQIP) is a population-based registry. The dataset, originally downloaded from Zhang et al. (PMC7498000) (32) under Creative Commons Attribution License, was sourced from the ACS NSQIP registry, encompassing a random sample of inpatients and outpatients undergoing non-trauma surgery in approximately 400 U.S. academic and community hospitals. This publicly accessible dataset enabled our secondary analysis while maintaining compliance with copyright regulations.

### Cohort definition

In our investigation, we downloaded the original database from Zhang et al. (PMC7498000) and analyzed a comprehensive set of variables, which were categorized into two distinct groups: continuous and categorical. The continuous variables encompassed anthropometric measurements (height and weight), preoperative laboratory parameters (hematocrit [HCT], serum sodium [Na], blood urea nitrogen [BUN], leukocyte count [WBC], serum creatinine [Cr], and platelet count [PLT]), along with surgical duration. The categorical variables comprised demographic factors (surgical year, gender, ethnicity, age category), comorbidities (diabetes mellitus, smoking status, functional health status, severe chronic obstructive pulmonary disease [COPD], congestive heart failure [CHF], medication-controlled hypertension), preoperative conditions (transfusion history, DC, systemic sepsis, chronic steroid use, significant weight loss exceeding 10% within six months, coagulopathies), and surgical characteristics (emergency status, wound classification). Body mass index (BMI) was computed using the standard formula (weight in kilograms divided by height in meters squared). This research, conducted under the approval of our institution’s Clinical Research Ethics Committee with exempt status, adhered to rigorous standardized protocols for data collection and processing. The exposure of interest was DC. Per ACS-NSQIP guidelines, patients with DC include those diagnosed with “cancer that has spread to one site or more sites in addition to the primary site”, or the “presence of multiple metastases which indicate the cancer is widespread, fulminant, or near terminal”. DC was recorded as a categorical variable (yes/no). An emergency case is usually performed within a short interval of time between patient diagnosis or the onset of related preoperative symptomatology. It is implied that the patient’s well-being and outcome is potentially threatened by unnecessary delay and the patient’s status could deteriorate unpredictably or rapidly. The definition of a patient with hypertension is that the patient’s condition is severe enough that it requires antihypertensive medication (for example, diuretics, beta blockers, ACE inhibitors, calcium channel blockers). Report the level of functional health status as defined by the following criteria. (1) Independent: The patient does not require assistance from another person for any activities of daily living. This includes a person who is able to function independently with prosthetics, equipment, or devices. (2) Partially dependent: The patient requires some assistance from another person for activities of daily living. This includes a person who utilizes prosthetics, equipment, or devices but still requires some assistance from another person for ADLs. (3) Totally dependent: The patient requires total assistance for all activities of daily living. (4) Unknown: If unable to ascertain the functional status prior to surgery, report as unknown. The detailed definitions of other variables can be found at the website: https://www.facs.org/media/kanfv4dl/ug12.pdf.

### Outcome

The primary outcome measure was postoperative 30-day mortality, defined as death occurring within the initial month following surgical discharge.

### Statistical analyses

This study included 18,642 participants. The dataset exhibited missing values for several key parameters: BMI (height and (or) weight) (n=730, 3.92%), BUN (n=1532, 8.22%), serum creatinine (n=709, 3.8%), WBC count (n=592, 3.18%), hematocrit (n=440, 2.36%), and platelet count (n=579, 3.11%). To handle missing covariate data, we implemented multiple imputation techniques based on the assumptions of missingness at random (33). Multiple imputation was performed using Empower Stats (X & Y Solutions, Boston, MA, USA) and R software (http://www.R-project.org, The R Foundation). The imputation model incorporated a comprehensive set of clinical variables, including anthropometric measurements (BMI), laboratory parameters (HCT, Na, BUN, WBC, Cr, PLT), surgical characteristics (procedure duration, emergency status, wound classification), demographic factors (age, gender, ethnicity), comorbidities (diabetes, COPD, CHF, hypertension), and preoperative conditions (sepsis, steroid use, significant weight loss, bleeding disorders). This approach ensured robust handling of missing data while maintaining the integrity of the statistical analysis. A 1:1 greedy matching algorithm without replacement was applied, with a caliper width set at 0.01. Although attempts were made to identify a narrower caliper, 0.01 provided the optimal balance. After multiple interpolations, 5 data sets were generated. Continuous variables were presented as mean ± standard deviation for normally distributed data or median (interquartile range) for non-normally distributed data. Categorical variables were expressed as frequency (percentage). Statistical comparisons between groups were performed using the Student’s t-test for normally distributed continuous variables, chi-square test for categorical variables, and Wilcoxon rank-sum test for non-normally distributed continuous variables.

To address potential confounding factors between patients with DC and those without DC, propensity score matching (PSM) was implemented to establish balanced cohorts (**Table 1**). The propensity score (PS) for DC was calculated using a comprehensive multivariate logistic regression model, with DC as the dependent variable and all baseline characteristics as independent covariates (34). The covariates included in the model are detailed in **Table 1**, encompassing anthropometric measures (BMI), laboratory parameters (HCT, Na, BUN, WBC, Cr, PLT), surgical factors (duration of surgery, emergency case, wound classification), demographic variables (gender, ethnicity, age group), comorbidities (diabetes, COPD, CHF, hypertension), preoperative conditions (systemic sepsis, steroid use, significant weight loss >10% in the preceding 6 months, bleeding disorders), and lifestyle factors (smoking habits, functional health status).

**Table 1:** Baseline characteristics before and after propensity-score matching in the original cohort.

Characteristic	Before Matching			After Matching		
	Non-disseminated cancer	Disseminated cancer	SD (100%)	Non-disseminated cancer	Disseminated cancer	SD (100%)
N	14620	4022		3653	3653	
BMI	29.009 ± 6.746	27.440 ± 6.467	23.7	27.838 ± 6.141	27.678 ± 6.504	2.5
Na	138.843 ± 3.141	137.807 ± 3.408	31.6	137.934 ± 3.542	137.954 ± 3.319	0.6
BUN	15.622 (12.000-20.000)	18.000 (13.000-24.000)	31.1	17.000 (13.000-23.000)	18.000 (13.000-23.000)	<0.1
Cr	0.800 (0.700-0.980)	0.800 (0.660-0.960)	2.5	9.300 (6.900-12.800)	9.600 (7.000-12.800)	1.7
WBC	8.200 (6.300-11.200)	9.800 (7.100-13.100)	29.8	9.300 (6.900-12.800)	9.600 (7.000-12.800)	1.7
HCT	40.875 ± 4.544	38.451 ± 5.271	49.3	39.086 ± 5.125	38.823 ± 5.073	5.2
PLT	235.000 (194.000-282.000)	239.000 (190.000-300.000)	12.8	240.000 (191.000-290.000)	237.000 (189.000-295.000)	2.9
Operating time	197.000 (131.000-287.000)	140.000 (97.000-196.000)	59	146.000 (100.000-206.000)	143.000 (100.000-202.000)	2.7
Gender			5.2			3.2
Male	7012 (47.962%)	1824 (45.351%)		1694 (46.373%)	1636 (44.785%)	
Female	7608 (52.038%)	2198 (54.649%)		1959 (53.627%)	2017 (55.215%)	
Race			8.9			10
White	10441 (71.416%)	2849 (70.835%)		2581 (70.654%)	2575 (70.490%)	
Asian	459 (3.140%)	84 (2.089%)		98 (2.683%)	79 (2.163%)	
Black or African American	925 (6.327%)	320 (7.956%)		208 (5.694%)	291 (7.966%)	
Unknown	2795 (19.118%)	769 (19.120%)		766 (20.969%)	708 (19.381%)	
Age ranges (years)			43.9			29
18-40	2822 (19.302%)	235 (5.843%)		395 (10.813%)	225 (6.159%)	
41-60	6038 (41.300%)	1704 (42.367%)		1315 (35.998%)	1584 (43.362%)	
61-80	5268 (36.033%)	1965 (48.856%)		1680 (45.990%)	1748 (47.851%)	
> 81	492 (3.365%)	118 (2.934%)		263 (7.200%)	96 (2.628%)	
Diabetes, N (%)	1690 (11.560%)	490 (12.183%)	1.9	469 (12.839%)	442 (12.100%)	2.2
Smoking status, N (%)	12198 (83.434%)	2834 (70.462%)	31.2	2689 (73.611%)	2675 (73.227%)	0.9
Functional health status			12.1			6.3
Independent	14029 (95.958%)	3757 (93.411%)		3458 (94.662%)	3432 (93.950%)	
Partially Dependent	452 (3.092%)	220 (5.470%)		144 (3.942%)	184 (5.037%)	
Totally Dependent	68 (0.465%)	26 (0.646%)		32 (0.876%)	22 (0.602%)	
Unknown	71 (0.486%)	19 (0.472%)		19 (0.520%)	15 (0.411%)	
Severe COPD, N (%)	355 (2.428%)	480 (11.934%)	37.5	273 (7.473%)	320 (8.760%)	4.7
Congestive heart failure, N (%)	39 (0.267%)	20 (0.497%)	3.7	14 (0.383%)	17 (0.465%)	1.3
Hypertension	5423 (37.093%)	1693 (42.093%)	10.2	1548 (42.376%)	1522 (41.664%)	1.4
Steroid use for a chronic condition, N (%)	1769 (12.100%)	1030 (25.609%)	35.1	846 (23.159%)	856 (23.433%)	0.6
>10% loss body weight in last 6 months	190 (1.300%)	215 (5.346%)	22.7	118 (3.230%)	144 (3.942%)	3.8
Preop Transfusions	38 (0.260%)	25 (0.622%)	5.5	21 (0.575%)	21 (0.575%)	<0.1
Bleeding disorders	240 (1.642%)	133 (3.307%)	10.7	106 (2.902%)	111 (3.039%)	0.8
Preoperative systemic sepsis, N (%)	434 (2.969%)	238 (5.917%)	14.3	182 (4.982%)	188 (5.146%)	0.7
Emergency case	823 (5.629%)	375 (9.324%)	14.1	342 (9.362%)	330 (9.034%)	1.1
Wound classification (Clean)	14207 (97.175%)	3920 (97.464%)	1.8	3568 (97.673%)	3556 (97.345%)	2.1

The adequacy of matching was evaluated by calculating standardized differences (SD) for all covariates at baseline, with a threshold of <10% considered indicative of satisfactory balance (35, 36). The discriminative ability of the logistic regression model was assessed using the C-index. The models were compared and selected via AIC. All analyses were conducted and reported in accordance with the STROBE guidelines (37).

For sensitivity analysis, the full dataset was used to construct multivariable logistic regression models. Additionally, to evaluate the potential impact of unmeasured confounding on the association between DC and postoperative 30-day mortality, E-values were computed (38).

Statistical analyses were performed using Empower Stats (X & Y Solutions, Boston, MA, USA) and R software (http://www.R-project.org, The R Foundation). Odds ratios (ORs) with corresponding 95% confidence intervals (CIs) were computed to quantify associations. A two-tailed p-value threshold of <0.05 was established as the criterion for statistical significance.

## Results

### Study population

**Figure 1:**
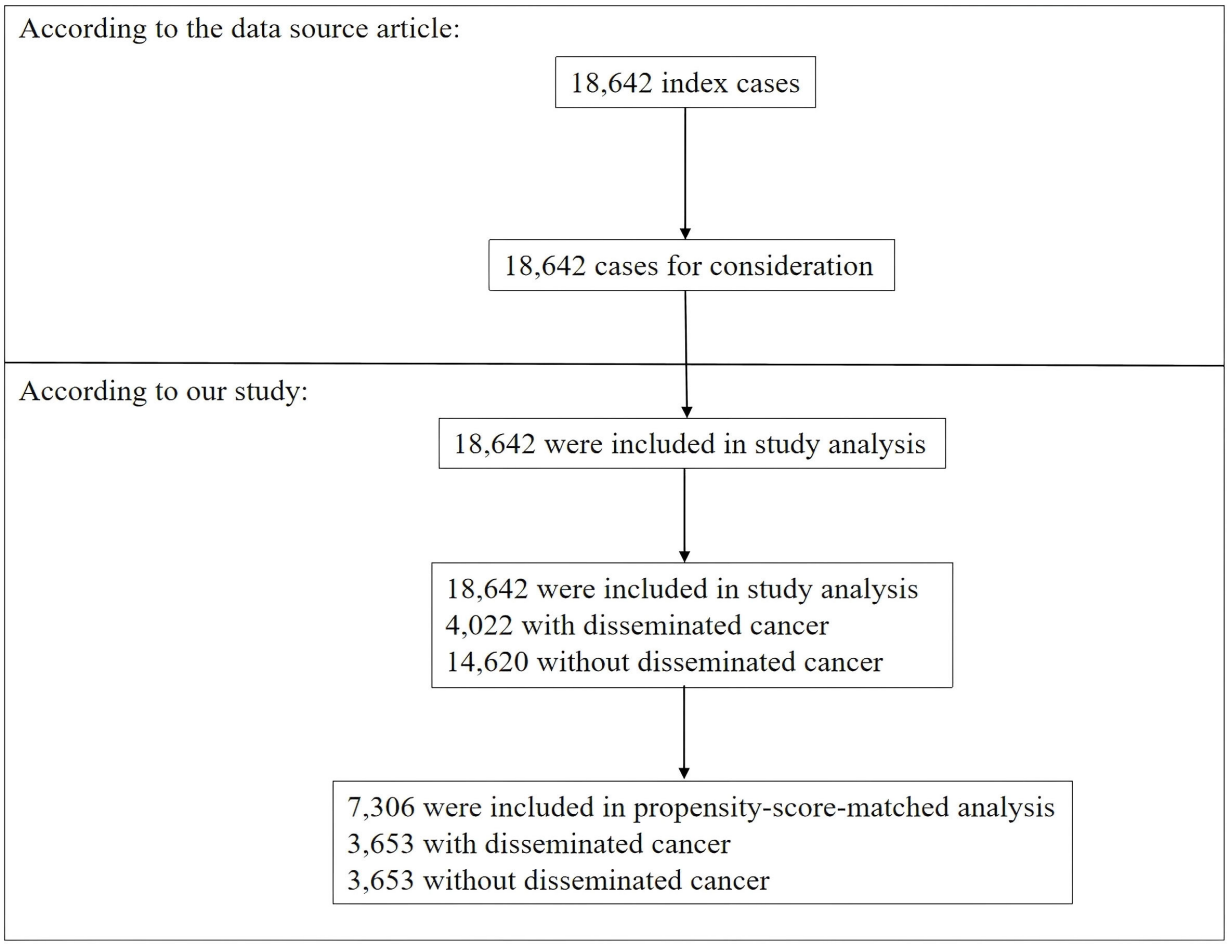
Flowchart of study participants.

The study included a total of 18,642 participants who satisfied the inclusion criteria (**Figure 1**). Of these, 52.6% were males, while the remaining 47.4% were females. Among them, 4,022 (21.57%) with DC, while 14,620 (78.43%) without DC. The P30d mortality were 4.97% (DC group) vs 1.77% (Non-DC group). Prior to PSM, there were variations in several fundamental attributes among individuals in the DC and without DC categories (**Table 1**). We found that individuals with DC generally had higher BUN, WBC, and PLT levels. With the use of one-to-one PSM, 3,653 patients with DC were matched with 3,653 patients without DC (**Figure 1**). Following the matching process, the majority of variables (excluding race and age ranges) exhibited standardized differences of less than 10.0%, suggesting successful matching of propensity scores. Specifically, there were minimal disparities in fundamental attributes between the two cohorts.

### Association between DC and mortality within 30 days after surgery

In the propensity score-matched cohort, we utilized a binary logistic regression model to examine the relationship between DC and postoperative 30-day mortality. The results were presented for unadjusted, minimally adjusted, fully adjusted, and propensity score-adjusted analyses (**Table 2**). In the basic model, DC was significantly associated with postoperative 30-day mortality (OR = 1.75, 95%CI 1.35–2.25, P < 0.001). Specifically, patients with DC had a 75% higher risk of postoperative 30-day mortality compared to those without DC. After minimal adjustments for gender, race, and age groups, the strong association remained significant (OR = 1.88, 95%CI 1.45–2.44, P < 0.001). Following comprehensive adjustments for various covariates, including gender, ethnicity, age, body mass index, HCT, Na, BUN, white blood cell WBC count, Cr, (PLT) count, duration of surgery, diabetes, smoking status, functional health status, severe COPD, CHF, preoperative transfusions, hypertension requiring medication, preoperative systemic sepsis, steroid use for chronic illness, significant weight loss (>10% in the past 6 months), bleeding disorders, emergency case, and wound classification, a significant correlation remained (OR = 2.06, 95%CI 1.57–2.70, P < 0.001). In the propensity score-adjusted model, the likelihood of postoperative 30-day mortality was increased by 72% in patients with DC (OR = 1.72, 95% CI 1.33–2.22, P < 0.001). These findings indicate that patients with DC prior to craniotomy are at a higher risk of postoperative 30-day mortality.

**Table 2:** Associations between DC and postoperative 30-day mortality in different models.

	Variable	Crude model (OR,95%CI, P)	Model I (OR,95%CI, P)	Model II (OR,95%CI, P)	Model III (OR,95%CI, P)
PSM cohort	Non-DC	Ref.	Ref.	Ref.	Ref.
	DC	1.75 (1.35, 2.25) <0.001	1.88 (1.45, 2.44) <0.001	2.06 (1.57, 2.70) <0.001	1.72 (1.33, 2.22) <0.001
Full cohort	Non-DC	Ref.	Ref.	Ref.	
	DC	2.91 (2.41, 3.52) <0.001	2.65 (2.19, 3.21) <0.001	1.82 (1.47, 2.26) <0.001	

Crude model: We did not adjust for other covariates.

Model I: We adjusted for gender, race, and age ranges.

Model II: We adjusted for variables such as gender, ethnicity, age group, BMI, HCT, Na, BUN, WBC count, Cr, PLT count, duration of surgery, presence of diabetes, smoking habits, overall health condition, severe COPD, CHF, presurgery blood transfusions, presence of hypertension necessitating medication, presurgery systemic sepsis, use of steroids for chronic conditions, weight loss exceeding 10% in the past 6 months, bleeding disorders, emergency cases, and wound classification.

In Model III, we adjusted for the propensity score.

The four analysis for PSM cohort included 7306 participants (3,653 with DC and 3,653 were not).

The three analysis for full cohort included 18642 participants (4,022 with DC and 14,620 were not).

Hazard ratios (HRs), confidence intervals (CIs), and reference categories (Ref) are the terms used in this context.

### Sensitivity analysis

After multiple imputations were employed, three model were conducted to assess the association between DC and postoperative 30-day mortality in the 18642 individuals. According to the full model, the DC group had a 82% greater risk of postoperative 30-day mortality than the group without DC in the original cohort (OR = 1.82, 95%CI 1.47-2.26, P < 0.001) (**Table 2**), which basically agrees with the results (OR = 1.72, 95%CI 1.33-2.22, P < 0.001) after PSM.

Furthermore, we computed an E-value to gauge the potential influence of unobserved confounding factors. This E-value was determined to be 3.53. The calculated E-value was found to be higher than the relative risk associated with unmeasured confounders and direct causes, suggesting that the relationship between direct causes and Postoperative 30-day mortality was scarcely affected by unmeasured or unrecognized confounders.

## Discussion

DC patients, their families and doctor, often struggle to make a choice between surgical treatment and conservative therapy due to the possibility of rapid deterioration of the condition if no surgery is performed, as well as the risks associated with the surgery for the patient. This study revealed a clear association between DC and postoperative 30-day mortality in adult patients with tumor craniotomy. The correlation exists both in the original cohort and the propensity-matched cohort. Compared with patients who underwent craniotomy for tumor resection without DC, our findings suggested a significantly increased risk of postoperative 30-day mortality in the population with DC. The authors hypothesize that the underlying reason may be that in patients with DC, multiple organs and systems may have been compromised preoperatively, leading to diminished overall resistance and immune competence. Since craniotomy for tumor resection constitutes a significant iatrogenic trauma, these patients’ tolerance for the surgical insult is reduced. This confluence of factors is likely responsible for the increased postoperative mortality observed in this patient population.

Neurosurgical intervention is likely to continue as a cornerstone of management of brain DC patients. The craniotomy is typically performed for single symptomatic metastatic lesions (39). Generally, surgical resection can help to obtain pathological diagnosis results which guide the formulation of subsequent treatment plans, alleviate the mass effect of tumors, and improve the quality of life of patients (11, 12). However, surgical intervention for patients with DC, even aiming to improve the quality of life of patients and symptomatic palliation, comes with substantial morbidity and mortality. Of 167,474 patients with DC, 50,669 (30.3%) were reported to have died within 30 days after index surgery (40). Some have questioned the role of surgery because of high surgical morbidity and mortality (41–43). Several preoperative laboratory indicators, such as hypernatremia, increased serum creatinine, and thrombocytopenia, independently predicted worse prognosis for metastatic brain tumor resection patients (44). Machine learning models were able to accurately predict postoperative 30-day mortality among a heterogenous population of DC (40). Our previous study reported DC as one of the important predictors of a prognostic model for predicting postoperative 30-day mortality after craniotomy in patients with brain tumors (21). In the current study, of 18,642 patients who underwent craniotomy for tumors, 4022 (21.57%) were DC patients, and the postoperative 30-day mortality in DC group was meaningful higher than non-DC group. Our results suggested compared with patients without DC who underwent craniotomy for brain tumor resection, patients with DC had a 72% to 106% increased postoperative 30-day mortality. To summarize, surgeons should pay special attention to the adverse mortality risk of craniotomy while recognizing the advantages of craniotomy for patients with DC. We believe that before the craniotomy, patients with DC and their families need to be informed of the relatively high postoperative 30-day mortality, so that they can make a more thorough consideration and choice regarding the craniotomy. If a DC patient needs to undergo a non-emergency craniotomy, his or her preoperative physical condition and risk factors should be comprehensively managed based on multidisciplinary collaboration to ensure a safe perioperative period. Postoperatively, close monitoring and prompt treatment should be carried out to reduce the postoperative 30-day mortality rate of such patients.

Our study boasts several distinct advantages that warrant recognition. To the researchers’ best recollection, there has been a scant number of retrospective analyses that have employed PSM to investigate the link between DCs and Postoperative 30-day mortality in adult patients undergoing craniotomy for tumor resection. PSM is a statistical technique designed to balance covariates at baseline to mitigate the influence of measured confounders. To validate the integrity of our results, a comprehensive sensitivity analysis was conducted. Additionally, our sample size was appreciably large, and participants were drawn from multiple centers, setting it apart from the majority of previous comparable studies.

However, it is essential to acknowledge the limitations of our investigation. Primarily, the study’s reliance on secondary data from published sources prevented us from entirely eradicating the impact of residual and/or unmeasured confounders that could affect the inferred relationship. Examples of such confounders include diverse factors such as dietary habits, socioeconomic status, pharmacological interventions, the characteristics of both neoplastic and non-neoplastic tissues, and the varied types and locations of DCs and brain tumors. The database employed in our research lacked neuro-oncologic variables and comprehensive information on certain treatments for DCs, such as pharmacological interventions and radiotherapy. Despite this, we calculated the E-value to gauge the potential influence of these unmeasured factors. The E-value of 3.53 suggests that an unmeasured confounder would need to have an implausibly strong association (≥3.5-fold with both DC status and postoperative mortality) to fully explain the observed effect. This supports the robustness of the findings; however, the interpretation requires appropriate clinical contextualization due to NSQIP’s lack of tumor-specific and neuro-oncologic variables. The study drew on a heterogeneous and sizeable dataset from individuals diagnosed with brain tumors, ensuring that the observed association and other findings remain credible and highly plausible. Future research should aim to collect and evaluate the impact of the aforementioned potential confounders and pertinent information that were not included in the present investigation.

## Conclusions

This retrospective study using individualized PSM revealed that DC was a significant factor influencing 30-day mortality following craniotomy for tumor resection in adult patients in the U.S. In patients with DC and high propensity scores, the risk of 30-day mortality increased by 72% to 106% compared to those without DC and low propensity scores. While recognizing the advantages of craniotomy for patients with DC, patients and their families should be informed of the relatively high postoperative 30-day mortality before the craniotomy, so that they can make a more thorough consideration and choice regarding the craniotomy. If a craniotomy cannot be avoided, then preoperative risk factor management, postoperative close monitoring and prompt intervention based on multidisciplinary collaboration are extremely important to reduce the high 30-day postoperative mortality rate of such patients.

## Data Availability

The original contributions presented in the study are included in the article/supplementary material. Further inquiries can be directed to the corresponding authors.
